# Book Notices

**Published:** 1869-12

**Authors:** 


### Book Notices.

Annual Report of the Board of Regents of the Smithsonian Institute, for the year 1868.

This is an octavo volume of 473 pages, filled with matter of much interest to men of science, and very valuable for reference.

Published at the Government Printing house in Washington, D.C.

Diseases and Injuries of the Eye: Their Medical and Surgical Treatment. By George Lewson, F.R.C.S. Surgeon to the Royal London Ophthalmic Hospital, Moorfields; and Assistant-Surgeon to the Middlesex Hospital. Philadelphia: Lindsey & Blakiston. 1869. Pp. 236. Price, $2.50. For sale by W. B. Keen & Cooke, Chicago.

This work is designed as a manual or text-book for students and general practitioners. It presents a convenient summary of the present status of Ophthalmic Science, in regard to the diagnosis and treatment of diseases of the eye. From the hasty glance we have taken of its contents, we think it will be found a very convenient and useful work.

---

The Pathology and Treatment of Stricture of the Urethra and Urinary Fistula. By Sir HENRY THOMPSON, F.R.C.S., Surgeon Extraordinary to H.M. the King of the Belgians; Professor of Clinical Surgery, and Surgeon to the University College Hospital. From the third and Revised London Edition. With Illustrations. Philadelphia: HENRY C. LEA. 1869. For sale by W. B. KEEN & COOKE, Chicago.

This is a well executed volume of 359 pages, presenting a full consideration of the subjects Urethral Strictures, and Urinary Fistulas, by an author of eminence and large practical experience.

---

A Handy-Book of Ophthalmic Surgery, for the use of Practitioners. By JOHN Z. LAURENCE, F.R.C.S., (University London), Surgeon to the Ophthalmic Hospital, Southworth; Ophthalmic Surgeon to St. Bartholomew's Hospital, etc., etc., etc., etc. Assisted by ROBERT C. MOOR, late Assistant-Surgeon to the Ophthalmic Hospital, Southworth. With numerous illustrations. Second Edition. Revised and enlarged. By J. Z. LAURENCE. Philadelphia: HENRY C. LEA. 1869. Pp. 227. For sale by W. B. KEEN & COOKE.

This is an improved edition of a well-known work, and will be found useful in every physician's library.

